# Exploring Factors That Influence Differential Attainment in MRCPsych Passing Rates in Resident Doctors in Core Training Based on Their Place of Qualification

**DOI:** 10.1192/bjo.2025.10300

**Published:** 2025-06-20

**Authors:** Prathibha Rao

**Affiliations:** Cumbria Northumberland Tyne and Wear NHS Foundation Trust, Newcastle, United Kingdom

## Abstract

**Aims:** Differential Attainment (DA) based on place of qualification has been demonstrated with consistent trends in Psychiatry. Factors contributing to this however, are ill understood. A significant proportion of residents in core training in Psychiatry are International Medical Graduates (IMG). Retention and consultant recruitment continue to be ongoing challenges in Psychiatry. We aimed to explore the awareness amongst trainers and to explore factors that influence DA, in relation to passing rates in MRCPsych examination, between the groups of IMGs and UK Medical Graduate (UKMG) resident doctors in core training.

**Methods:** Following a constructivist ontology, eight semi-structured interviews were conducted with core psychiatry trainers who were recruited via purposive sampling technique. Interpretative phenomenological analysis was used to identify themes.

**Results:** DA in the CASC diet of the MRCPsych examination is known but not the extent of this. Trainers are not aware of DA within theory papers. Five themes were identified that influence DA: Trainee factors, examination factors, trainer factors, training factors and the relationship between trainer and trainee. Greater emphasis for communication in exams along with difficulties in communication within IMGs were identified as the most important factors driving DA. Understanding the nuances of cultural differences and mastering exam technique requires time, and IMGs may feel unprepared but feel pressured to follow the same progression timelines as their UKMG counterparts. Trainers are well placed to address this within supervision early in the training, but competing priorities to cover within supervision, along with the sensitivity of the topic and fears of being perceived negatively pose as barriers. Misinformation and lack of awareness of extent of DA within trainers also contribute to this. Unfamiliarity with exam technique amongst trainers combined with residents perceiving exams as being personal rather than a shared objective of training, affects this being actively addressed within supervision.

**Conclusion:** Implementing practical, person-centred and consistent measures to support equity rather than equal opportunities is key. Communication skill courses are well embedded within training programmes, but trainers need to be supported in enhancing sociolinguistic competence of individuals rather than just improving language. Involving all core trainers in local CASC revision sessions and encouraging them to be CASC examiners will improve their confidence in supporting residents. Raising awareness of DA with both groups of trainers and residents whilst making simultaneous efforts to understand and tackle unconscious bias, improving cultural intelligence and reinforcing trainer-trainee relationship will create a safe space to address this sensitive yet important issue.

